# Is Procreation Special?

**DOI:** 10.1007/s10790-021-09797-y

**Published:** 2021-02-17

**Authors:** Ingrid Robeyns

**Affiliations:** Ethics Institute, Utrecht University, Janskerkhof 13, 3512BL Utrecht, The Netherlands

## Introduction

The current ecological crisis shows that ecological resources, in particular the capacity of the atmosphere to absorb greenhouse gas emissions, are scarce: all humans want to use more ecological resources than is sustainable, and in fact are doing so.^1^ Since we all need these ecological resources to live our lives well, and since, pre-institutionally, they cannot conceivably be understood as the property of a specific person, the allocation and use of ecological resources are deeply political and hence a matter of distributive justice. But how should we deal with the fact that those among whom the ecological resources are to be fairly distributed are simultaneously the creators of new lives and hence the creators of additional human beings, among whom those scarce ecological resources should be fairly distributed? Should it be morally permissible for a person who refrains from procreating to expend the emissions that they prevent by not procreating on additional consumption activities? Is a person who has no children and claims that therefore he is morally permitted to emit more on these other activities than parents justified in making this claim?

In this paper, I make an attempt to make progress in analyzing the status of procreation in the question of the fair distribution of resources, in particular environmental resources. If we aim to reduce our impact on ecosystems in order to reach an ecologically sustainable equilibrium, should we consider procreation to be morally equivalent to overconsumption (whereby overconsumption is understood as the consumption of goods and services beyond those needed to meet one’s basic needs)? The answer to this question has implications for the debates on fair emissions quotas or permits, because whether the emissions caused by procreation should be taken from the parents’ emissions quota or not depends in part on the question of whether emissions are seen as morally on a par with consumption and other forms of emitting acts.^2^ If procreation and overconsumption are morally equivalent, then one’s fair emissions share would not increase if one wanted to procreate. Instead, the emissions needed to procreate and parent would come from the same individual emissions budget. If two persons have the same individual emissions budget before either of them has children, the person who procreates will not be given additional emissions rights. A nonparent could claim that it is morally permissible for him to consume more than a person who procreates, since he is opting to not take those ecological resources that procreation would require. The nonparent could claim that as long as his total appropriation of ecological resources does not exceed that of the parents and the offspring those parents choose to have, and as long as he is not exceeding his personal quota, there is no case of unfairness. After all, if a parent’s procreating acts require additional natural resources, why shouldn’t a nonparent be morally permitted to expend the same amount of natural resources in a different way, for example via traveling extensively or building a private swimming pool?

This idea—that in matters of (environmental) distributive justice, procreation and consumption are equivalent—is the dominant view among philosophers who have written on this question. It has been defended explicitly by Thomas Young and by Corey MacIver, and is, either implicitly or explicitly, endorsed by most liberal philosophers who have published on this topic.^3^ In contrast to the work of these authors, in this paper, reasons will be given for why we should not endorse the Moral Equivalence Thesis regarding matters of (environmental) distributive justice.^4^

In the next section, the case of Ann and Pearl is described. Ann is happy to forfeit her right to procreation so she can stick to a life of greater affluence, including overconsumption, whereas Pearl has children. Can Ann expend more ecosystem resources on consumption than Pearl? As was just mentioned, an influential way to answer this question is to adopt the liberal equality of resources view, which is analyzed and criticized in section 3. An alternative way of answering this question is developed next that draws on an account of human flourishing that is based on the capabilities approach (section 4). A capabilitarian view allows us to reject Ann’s claim to more ecosystem resources, but at the same time limits the procreative freedom of Pearl (section 5). This brings us to the first critique of the view that procreation and consumption are morally equivalent, namely that it is based on an implicit acceptance of resources as the metric of distributive justice. If, instead, we endorse capabilities as the metric of distributive justice, we do not necessarily have to accept that claim. In section 6, how one could develop a second and independent argument is sketched out—this is the argument from human self-understanding. This argument moves beyond the terrain of liberal theories of distributive justice. Instead, it draws on the claim that we should conceptualize procreative parenting as part of our self-understanding. If the argument from human self-understanding is correct, then it leads to a rejection of the view that procreation and consumption are morally equivalent. In the final section, an objection is briefly addressed, and some conclusions are drawn.

Before these arguments are presented, three caveats are in order. First, this paper is a contribution to the literature on the status of procreative decisions when we are considering questions of distributive justice, in particular environmental distributive justice.^5^ Therefore, the only question that is addressed here is whether procreation is special in the context of distributive justice, hence when we are trying to answer questions such as who should bear the cost of particular acts and the amount of resources that should be allocated to various persons. The related but distinct question of what makes procreation morally permissible in the first place is not addressed in this paper. That question belongs to a cluster of questions about the value of procreation and the family, what specific moral rights procreators and parents should (not) have, and which feature of procreative parenting would make it valuable relative to adoptive parenting (gestation, genetics, love, or particular relationships). This is a somewhat different literature.^6^ Not surprisingly, these two literatures are to some extent related, and in this paper it will be shown that the distributive justice literature could benefit from incorporating some insights from the philosophical literatures on the value of procreation and the family. Yet the argument presented in this paper should be read as a contribution to the debates in the former literature, rather than the latter.

Second, in the environmental distributive ethics literature, the word procreation is used as shorthand for procreative parenting. In contrast, in the literatures on the value of procreation and the family, much attention is devoted to the distinctions between procreation, procreative parenting, and parenting. In section 7, I will turn to those concerns, yet will argue there that they do not have significant implications for the assessment of the Moral Equivalence Thesis.

Third, the Moral Equivalence Thesis is an abstract philosophical principle, not a rule for direct implementation in society. There are many normative hurdles that have to be overcome from the point of accepting an abstract philosophical principle to implementing it. For example, there might be overruling considerations, or there might be motivational or practical feasibility constraints that make immediate implementation impossible. An important hurdle relating to implementation could be that the moral cost of enforcing the principle is too high and therefore the principle could only be justified as a pre-political or pre-institutional moral principle that people could (and morally should) take into account in their own deliberations, but not as an institutional principle. The distinction between abstract philosophical principles and the rules for implementing them is obviously very relevant for the Moral Equivalence Thesis, and this is only analyzed in this paper at the level of theoretical principles.^7^ Is a solid critique of the Moral Equivalence Thesis at the level of abstract theoretical analysis possible?

## The Moral Equivalence Thesis

Ann and Pearl are two women of the same age who live in the same neighborhood. They both have well-developed mental, moral, and cognitive capacities. Both can be considered moral agents and can be attributed moral responsibility for the decisions they make. Ann enjoys her affluent lifestyle and rejects the option of being a procreator: she does not have children and has opted to be sterilized to make sure she will never become pregnant. Pearl, on the other hand, is a procreator: she has two children and has not yet decided whether or not to have more. Given what we know about the negative impact of procreation on issues of ecological sustainability, this implies that with the same consumption pattern, Pearl’s impact on the ecosystem will be significantly larger than Ann’s. The question is whether Ann should be morally permitted to increase her impact on the ecosystem by changing her material lifestyle up to the level she would have had if she also had two children. For example, Ann could fly on a more regular basis, eat more dairy, build a heated swimming pool in her house, or indulge in other forms of overconsumption. What should we think of Ann’s claim that she is entitled to have the same level of impact on the ecosystem as Pearl and others who voluntarily have children?^8^ We can formulate this question differently: if we determine each person’s fair emissions quota, should procreators receive extra emission rights to cover the emissions needed to procreate and raise children, or should procreators instead use their own emission rights to cover the emissions of their offspring?

Ann’s claim has been defended by Thomas Young and Corey MacIver.^9^ Young argues that if one views overconsumption as morally problematic (as ecologists do), one must, in order to be consistent, also regard having children as equally morally problematic. Young reaches this conclusion via an argument based on analogical reasoning, which runs as follows. First, creating a new person is creating a set of claims on natural resources, and hence qua impact it is equivalent to other actions that entail a claim to such natural resources, such as consumption. Second, we morally condemn overconsumption because of the environmental harms it causes. Third, since there is no morally relevant distinction between overconsumption and procreation, we have to condemn procreation just as much (or as little) for the ecological harms it causes as we have to condemn overconsumption. As MacIver puts it, Young reaches the conclusion that “there is no morally relevant distinction between an individual claiming a large quantity of natural goods for her own personal use, and an individual claiming a large quantity of natural goods for the use of her offspring. There is nothing inherently special or defensible about the latter”.^10^

This is the Moral Equivalence Thesis in the debate on procreation and (environmental) distributive justice. The only way this thesis can break down is by showing that there is something special about procreation compared to other ways in which ecological resources are used beyond what is needed to meet our basic needs, that is, overconsumption. Young reviews several potential arguments that one could use to undermine the Moral Equivalence Thesis, yet he claims that none of them is convincing. While the Moral Equivalence Thesis has been defended explicitly by Young and MacIver, it is also implicitly assumed in the arguments of other philosophers, such as Peter Vallentyne, who argues for individual ecological resource entitlements (or emission quotas) whereby procreators should take the resources that their offspring need from their own entitlements, and Paula Casal and Andrew Williams, who argue that nonparents should not pick up the bill of the negative externalities that procreation causes.^11^ More recently, Simon Caney has defended a view he calls ecological liberalism, which also treats procreative choices as they relate to matters of environmental distributive justice as on a par with other choices, including choices concerning overconsumption.^12^

So far, no-one has questioned or criticized the Moral Equivalence Thesis directly at the level of abstract principle, although some more applied or policy-oriented papers on population and ecological unsustainability reject the Moral Equivalence Thesis, either implicitly or explicitly.^13^ Yet given that almost all distributive justice theorists who have written on procreation and environmental distributive justice endorse it, we have a strong reason to critically examine the Moral Equivalence Thesis at the level of abstract moral theories and principles. That will be the challenge taken up in this paper. Two critiques of the Moral Equivalence Thesis will be offered: a capabilitarian argument and an argument from human self-understanding. The former will show that the Moral Equivalence Thesis relies on an endorsement of resources as the metric of distribution justice, while the latter will lead to a rejection of the Moral Equivalence Thesis by showing that there is a morally significant difference between procreation and consumption that is relevant when we are deciding how to share scarce resources. But before we turn to those critiques, we must first analyze in more detail the arguments of defenders of the Moral Equivalence Thesis.

## Equality of Resources

Several resource egalitarians subscribe (implicitly or explicitly) to the Moral Equivalence Thesis. At the core of resource egalitarianism are two claims: (1) that distributive justice should be evaluated in the ‘evaluative space’ of resources, and (2) that the distribution of resources in society should be such that it is sensitive to people’s preferences, ambitions, and choices but not to those factors that are considered brute luck or beyond people’s control. That second claim is the characteristic that is needed to make a resourcist theory also a luck-egalitarian theory. Yet not all luck-egalitarian theories need to be resourcist; they could also look at matters of distributive justice in other dimensions, such as capabilities or utility. Resource-egalitarian theories of distributive justice contain many theoretical varieties, yet the subtle differences between these various forms are not relevant for the present analysis. What is relevant is that the factors (including actions) that have a causal effect on a person’s level of advantage must be categorized as either a preference (which some, following Ronald Dworkin, call an ambition) or a factor beyond one’s control (or “an endowment,” in Dworkin’s terminology).^14^ The equality of resources view holds that one is *prima facie* responsible for the costs of one’s ambitions or preferences but also holds that distributive justice requires compensation for what is called brute luck—the effects of those factors for which one can’t be held responsible, such as one’s endowments and the circumstances in which one finds oneself.

Within the framework of equality of resources, procreation is an ambition and hence a choice for which procreators can be held responsible and should, as far as distributive justice is concerned, bear the costs. Barring cases of pregnancies and births resulting from coercion, the lack of the necessary mental capacities for minimal agency, or the lack of the material means to prevent pregnancies, it is argued that procreators make a choice to try to procreate, or make a choice to engage in sexual intercourse without taking the necessary measures to avoid (or terminate) a pregnancy. The dominant view among resource egalitarians is that if certain minimal conditions of agency and voluntariness are met, procreators can be held responsible for the procreation, in the sense that they are the ones who should bear the costs of procreative acts and their consequences.^15^

The dominant application of the equality of resources view to the case of procreation is to argue that persons choose to have children and that this is a *pro tanto* reason for those procreators not to be compensated or accommodated for the consequences of their choice. There shouldn’t be measures such as parental subsidies to compensate for the cost of procreation.^16^ Given that pregnancies require sexual intercourse, and given that in the case of minimally autonomous agents we can safely assume that they know that sexual intercourse may lead to the woman becoming pregnant, and assuming a wide availability of contraceptives, people can be held causally responsible for the pregnancy and the baby that will subsequently be born.^17^ They may have deliberately desired and aimed for the pregnancy, or the pregnancy may have happened nondeliberatively, after which the procreators decided to continue the pregnancy. In either case, given the availability of contraceptives and abortion services, it is, if one reasons from within this framework, plausible to assume that pregnancies conceived under those circumstances should be regarded as resulting from the parents’ so-called ambitions or preferences. As a result, there is, according to resource-egalitarian theories, nothing special about procreation that warrants us to treat procreative preferences as different from preferences to drive a fancy car or drink expensive wine.

How should we evaluate this view? One problem with resource-egalitarian theories is that they don’t have the theoretical resources to even ask whether procreation is special. That is because their dichotomous theoretical framework forces us to categorize each factor affecting a person’s disadvantage as an ambition (or a preference) or a factor beyond one’s control. The distribution of resources will be sensitive to the latter, but not to the former. All factors that impose a cost (including nonmonetary costs and opportunity costs) on the relevant person, or on third parties, or on society at large have to be conceptualized as resulting from that person’s preference and ensuing choice or from a factor beyond her control.

But why should these be the only two categories that are relevant to studying actions that raise issues of distributive justice and fair divisions? Are these the right categories for our analysis? Underlying resources as the metric of distributive justice is the theoretical basis of two notions— preferences on the one hand, and factors beyond one’s control on the other. Yet there are two problems with this conceptual apparatus when we want to examine the Moral Equivalence Thesis. First, the notion of preferences is theoretically much less sophisticated than other notions such as capabilities or the distinction between different types of needs and wants. As is well known, the notion of preferences (which underlies resource egalitarianism) does not allow a distinction to be made between desires that are morally urgent and those that are not; as a consequence, it erases relevant moral distinctions when thinking about individual and social choices.^18^ Second, forcing all causal factors of the resulting outcomes into the choice-endowment dichotomy can’t be quite right.^19^ There are a number of factors that don’t fit this dichotomy, such as religious or ethnic affiliation, gender, or indeed parenthood. Those factors demand an analysis that the dichotomous distinctions between ambition and endowment and between option luck and brute luck don’t allow for.

Is it possible to modify and extend the equality of resources view to broaden its scope? This is exactly what Paul Bou-Habib and Serena Olsaretti have done: they have extended the Dworkinian hypothetical insurance scheme to second-order preferences of first-order preferences about the good life.^20^ Making use of a hypothetical insurance scheme that would provide funds for the costs of adequate child-rearing if we decide to have children, Bou-Habib and Olsaretti make the argument that the state must provide parental subsidies to the extent that individuals would, under suitable hypothetical conditions, choose to buy such insurance. While this proposal provides a justification for societal support for parents, it still imposes the Dworkinian distinction between choices and endowments upon our normative analysis. Procreative parenting is still understood as merely a choice that is based on one’s preferences—with the modification that it is now regarded as a choice that we find so important that we are willing to insure ourselves for the risk that we might want to become parents yet may not have enough resources to do so.

The argument of Bou-Habib and Olsaretti is very interesting, but it is quite different from the arguments advanced in this paper. The first difference relates to concepts, language, and style. In this paper, the question of the environmental justice of procreative parenting is analyzed using a different conceptual language and starting from a different methodology, which is less theory driven.^21^ Methodologically, this approach will make it possible to use the outcomes of this analysis for a subsequent step that moves from theoretical analysis of abstract principles to their consequences in the real world.^22^ Second, Bou-Habib and Olsaretti’s argument is a conditional argument: to the extent that people would be willing to hypothetically insure themselves to keep open the option that they would like to be a parent in the future, the modified luck-egalitarian account can justify parental subsidies. In this paper, by contrast, I bite the bullet, and develop an argument why people should be willing to keep open the option of being a parent when designing a just society and why distributive justice does not morally permit the nonprocreator to use more resources from the ecosystems through consumption. The argument in this paper gives a phenomenological account of why parenting is morally distinct from overconsumption and uses this in an argument about distributive justice. And third, the ultimate reasons for socializing some of the costs of procreative parenting are different. One could argue, as Bou-Habib and Olsaretti do, that combining equality of resources with hypothetical insurance will make people want to support procreative parenting. If this is applied to the case of ecological resources, it would imply that parents would not have to suffer lower levels of consumption because of their procreative choices. Similarly, in more recent work, Olsaretti has rejected the view that the costs of procreative parenting should not be shared between parents and nonparents. Olsaretti has developed an analysis within the framework of liberal egalitarianism, using resources as the appropriate metric.^23^ Yet it is disputed that matters of distributive justice should be decided using the distributive metric of resources. What difference would it make if one were to endorse another metric of justice, such as capabilities? That is what is investigated next, by first describing how the capability approach would conceptualize procreative parenting (section 4) and then asking what difference the capability approach makes for the plausibility of the Moral Equivalence Thesis (section 5).

## The Capability to Engage in Procreative Parenting

In order to assess the Moral Equivalence Thesis, we have to ask how we should, within theories of distributive justice, conceptualize procreative parenting. My suggestion is that we should consider procreative parenting as a valuable capability that should be part of the set of capabilities that form the metric of social justice. Capabilities are those beings and doings that we have genuine access to. If we are said to have a capability to X, this means that if we want to do X, we can do X. The doings and beings are called functionings; the corresponding opportunities are called capabilities.^24^ Procreative parenting is a capability *par excellence*, since it is both a way of being (one is a parent) and a doing (one engages in parenting). The capabilities that make up the metric of justice are those that, upon reflection, we find valuable. This reflection is not a matter of unreflective preferences for certain capabilities, but rather the outcome of a legitimate process of public reasoning and deliberation.

In the capability approach, well-being can be expressed in two dimensions: achieved well-being and the opportunity to achieve well-being—what Amartya Sen calls well-being freedom. The achieved well-being is the level of achieved functionings that a person has realized. Yet in most cases the relevant metric of justice is not achieved well-being but the opportunity to achieve well-being, which is a person’s set of valuable capabilities.

For the argument that follows, I will endorse the view that social justice should be conceptualized in the space of capabilities. The reasons why we should not be interested in resources per se but rather in the freedom to achieve well-being that resources generate have been discussed extensively in the capability literature.^25^ This, then, prompts the following question: should procreative parenting be a capability we should care about when we are considering what we owe each other? Is procreation so special that it would best be understood as a dimension of well-being freedom that is relevant for matters of justice?

Procreative parenting can be seen as both a valuable being and a valuable doing. Procreative parenting can be conceptualized as a capability that is not only valuable but also incommensurable with any other capability. From this perspective, people do not want to have a child in the same way that they want to travel or engage in a hobby: rather, they want to have a child because they want to be a mother or a father—to be the person to whom a child goes if the child falls and gets hurt and needs to be comforted. In many peoples’ lives, children are both the givers and the receivers of the most intense love. Those who want children because they value the capability of parenting want them because of the unique relationship that they expect children will give them: a relationship that is characterized by a unique emotional intensity, and generally also by unconditional love when they are young, and a deep love respecting the person they are becoming when they grow up.^26^ This relationship is also unique in its relation to the dependency of one person (the child) on the other person (the parent), which is complete at birth but decreases as the child develops. In addition, parenthood changes and matures one’s identity: being a mother or a father has implications for various domains of one’s life and drastically changes one’s priorities, the structure of one’s freedoms, and one’s responsibilities. Procreating and raising children thus provides a unique window onto life; it is an existential experience that is not experientially commensurable with other experiences.

The substance of the point I am making is not new; I am merely pointing out that one can see the valuable and unique relationship between parents and children as a capability, and that this conceptualization allows us to see how procreation and parenting fits into questions of distributive justice. Philosophers who have studied procreation from the perspective of the value of the family and the morality of having children have theorized about the value of parenthood using a range of terms that all point to these goods of the parent-child relationship.

For example, Colin Macleod argues that there are several ways in which the family is a source of value, some of which focus on the interests of the parents: the intimate relationships that parents have with their children; the identity that having children provides; and the care that parents may receive from their children when they age.^27^ Harry Brighouse and Adam Swift argue that the parent-child relationship has distinctive value that makes an important contribution to the flourishing of the parents. The relationship with children has an intimacy that is qualitatively different to other relationships, given the uncontrolled passions, emotions, and interests that children reveal to their parents. Moreover, parents have an interest in the fiduciary role they play for their children.^28^ Crucially, they conclude that parenting is an incommensurable relationship. According to Brighouse and Swift, “the intimacy one can have with one’s children is quite different from the intimacy one can have with other adults. It makes a contribution to one’s flourishing of a different kind, and, for many, is not substitutable by relationships of other kinds”.^29^ Christine Overall also highlights the uniqueness of the kind of relationship that occurs due to procreative decisions, and stresses that this relationship is key to understanding what is valuable about procreation.^30^ Luara Ferracioli, extending Overall’s views, has developed an account of the value of procreative parenting based on the deepness and robustness of the love between parents and children. Her account also highlights how the love between a procreator and the child is distinct from other types of relationships, such as between friends or lovers.^31^ Rivka Weinberg stresses that procreation should be understood as an act by adults who want to enrich their lives by engaging in the parent-child relationship.^32^ While each of these authors is asking the question what is valuable about procreation as part of slightly different philosophical projects, they all point to the central importance of the unique relationship between parents and children for understanding procreative parenting.

What do these arguments and insights from the literature on the value of procreation and the family imply for the status of procreation in (environmental) distributive justice? If we endorse capabilities as the appropriate metric of justice, we want to make sure we have fair opportunities for well-being, which requires that society is organized in such a way that the most important capabilities are secured. Procreative parenting is such a capability. If we are endorsing capabilities as the metric of distributive justice and are asking what a just society would look like, and we know that many people care deeply about procreative parenting and have very good reasons for doing so, then surely we would think that a just society should be a society in which the opportunity or capability to procreate would be protected, including in the sense that we shouldn’t have to pay an excessive price if we want to use that opportunity.

## Resources, Capabilities and the Moral Equivalence Thesis

What difference, if any, does it make if we see procreative parenting as a valuable capability rather than as a mere preference within the framework of resource egalitarianism? What difference does it make to the Moral Equivalence Thesis and to the assessment of Ann’s claim that she should be allowed to use resources from the ecosystems for various forms of overconsumption, since she is not procreating?

Under resource egalitarianism, every person would be given the same resources (with compensations for disadvantages that are not chosen, such as disabilities or weak innate talents). Each person would be given a fair allocation of nonrenewable resources, access to water, and rights to emit greenhouse gasses. Each person could choose what to do with their resources—they could consume them or pass them on to the children they would have. All preferences would be treated equally: no distinction would be made between preferences for valuable capabilities, preferences for idiosyncrasies, or excessive amounts of consumption that do not increase valuable capabilities. Given the significant impact on the ecosystem that procreative parenting has, resource egalitarianism in practice implies that parents would only be able to enjoy a fraction of the emissions-weighted consumption of nonparents, since parents would need to allocate part of their emissions entitlement to their children.

But under the capabilitarian view defended in this paper, that conclusion does not necessarily follow. Parents and nonparents alike should have equal access to a range of valuable capabilities, which includes the opportunity to have children. Ann has a right to the same access to certain levels of her capabilities as Pearl has. Her decision to not use one of those capabilities does not imply that she has a right to expend the resources from the ecosystems that she did not expend on procreation and parenting on capabilities that are not available to parents. From a capability perspective, in the context of great scarcity, we owe each other access to a minimal set of valuable capabilities. Which exact capabilities would be part of that minimal set would have to be decided by a democratic process that is legitimate and can be defended on moral grounds. That process should identify a set of capabilities that everyone should have access to.

However, what also doesn’t follow from the account of the value of procreative parenting developed in this paper is that from a moral point of view, people can have as many children as they please. If their procreative behavior (or other behavior) endangers the procreative capabilities of future generations, or if it endangers other valuable capabilities of their contemporaries or future people, then there will be moral limits to their procreative capabilities.^33^ The account of procreative parenting developed in this paper also explains why the contribution of a first child to one’s flourishing is much bigger than for subsequent children. This confirms and strengthens the argument made by Christine Overall, Sarah Conly and others that the moral justifications for creating a child (per couple or per person) are much stronger than justifications for additional siblings – if there are any at all, that is.^34^ With one child, many of the morally relevant aspects of parenthood described earlier are secured, such as the identity of being a parent, the intense love and special form of intimacy, the opportunity to be able to see the world through the eyes of an innocent new human being, and getting a unique perspective on life. With a second child, what is added is more limited: being a close witness to the relationship between the siblings and the possible new experiences introduced by the fact that siblings are often very different. Hence, the additional value of a second child to the flourishing of parents is much smaller than that of the first child; and it is plausible to assume that the additional value added to the parents’ flourishing decreases, even if only to a small degree, with every additional child.

We have to be very careful when asking what exactly follows for the Moral Equivalence Thesis. The capability approach to justice can be designed in many different forms, which will have implications for whether Pearl would have to share her own individual greenhouse gas emissions budget with a possible third child (perhaps even with her second child). A capability theory of the fair allocation of individuals’ emissions budgets could defend the view that everyone should be given an individual emissions budget that is needed to meet the basic capabilities; if the total of those individual emissions budgets is smaller than the aggregate emissions budget, the remaining budget could be divided up into a so-called luxury budget per capita. At that point, procreative decisions would be regarded as equivalent to decisions about overconsumption. This would be justified, since the valuable capability to engage in procreative parenting would be met up to a sufficiency level. However, depending on the remaining aggregate global emissions budget, it could also be the case that there won’t be any per capita luxury emissions budget to be divided up. In that case, each person would be allowed an emissions budget to meet their basic consumption needs and an emissions budget for one or two children per couple. There are climate change scenarios conceivable in which even the second child per couple would be violating principles of ecological sustainability and intergenerational justice. Under those scenarios, fair divisions may not give us more than the emissions budgets for meeting our basic consumption needs and at most for those of one child per couple, which would also require us to rethink social institutions of child-rearing in such a way that all adults can enjoy the goods of parenting and the needs of children are met.

Let us take stock. The capabilitarian argument against the Moral Equivalence Thesis, as presented here, does not reject the thesis. Rather, it shows that the acceptance of the Moral Equivalence Thesis depends on the prior acceptance of some version of the equality of resources view as the appropriate theory of distributive justice. If, instead, we endorse a capabilitarian theory of distributive justice, it is conceivable that a normative theory can be developed that does not endorse the Moral Equivalence Thesis. The full development of such a theory—and especially its justification—is beyond the scope of this paper. The more limited aim here has been to pave the way to showing how, within distributive justice, one can resist the Moral Equivalence Thesis by changing the metric of distributive justice and by conceptualizing procreative parenting not as a preference but as a valuable capability.

I will now develop a second critique of the Moral Equivalence Thesis. This second critique, the argument from human self-understanding, is both stronger and weaker than the capabilitarian argument. It is weaker because it does not analyze the (technical) differences between different philosophical theories, but instead is more speculative and based on a larger range of nonphilosophical sources. But in another sense it is also stronger, because, if true, it leads to a rejection of the thesis rather than a merely conditional rejection, as is the case for the first argument (which is conditional upon the acceptance of the capability approach as the theory of distributive justice rather than liberal egalitarianism).

## The Argument from Human Self-Understanding

Debates about social ethics are dominated by concerns relating to distributive justice as well as freedoms and rights. A full analysis in the domain of social ethics requires us to take other moral considerations into account. There may be institutions that maximize the value of distributive justice but that are nevertheless morally reprehensible in another aspect that is not captured by the distributive justice concerns. And another moral consideration is whether we are respecting the principle that we should be treated as human beings and should not be downgraded and treated as something else. When deciding how we should distribute scarce common resources such as emission rights, the correct distribution of the metric of justice is not the only thing that matters to social ethics; it also matters that we implement a distributive rule that is not dehumanizing. In order to know whether that is the case if we were to live in societies that have institutions that are based on the Moral Equivalence Thesis, we must first ask when a certain rule or institution would be seen as dehumanizing. This requires us to ask the difficult question of what it means to be human.

These are questions that belong to the realm of philosophical anthropology. However, theories of distributive justice make very few links to philosophical anthropology; moreover, there are methodological challenges in making the insights of philosophical anthropology fit into the detailed and fine-grained categories and distinctions used by analytical philosophers working on distributive justice. Yet philosophical anthropology can help us to develop the argument from human self-understanding that leads to a rejection of the Moral Equivalence Thesis. The starting point of the argument from human self-understanding is that human beings understand themselves not merely as rational agents who can act purposefully but also (though evidently not exclusively) as human animals. As animals, we are a procreating species. As humans, we engage in thinking, reasoning, arguing, being creative, caring about morality, and reinterpreting and giving meaning to urges that flow from the animal side of our being. We see ourselves not as robots or as disembodied souls, but rather as members of the human species—a species that procreates and has developed many social practices and traditions that give meaning to parenting and to kinship. Thinking of having a baby as merely an individual choice is misleading, because our procreative decisions take place against this context in which we understand ourselves as a member of a species that engages in parenting and kinship relationships as meaningful social practices and traditions. This membership is not only important for our identity but also so that we are able to see ourselves as humans that belong to humanity. The denial of this membership via the design of a social institution or policy is dehumanizing, and hence is a reason to morally condemn such an institution or policy.

We don’t have to endorse a specific theory of philosophical anthropology to see that having the opportunity to see ourselves, and to be treated, as truly human has implications for how we conceptualize procreation. The possibility of having and raising children is a universal and very deep aspect of our self-understanding. Before we procreate, our own lives are the last node in a long genealogy of procreating human beings. By procreating and raising children, we are adding a node and thereby connecting the past and the future and extending the genealogical tree. Having the opportunity to procreate is thus part of our self-understanding of being part of a humanity that not only has a past, but also a future. The continuity of the human species is needed to create the moral community in which we ask moral questions such as what we ought to do to preserve the Earth for the future. In contrast to nonhuman animals, we can think, reason, and deliberate about our procreative behavior. This implies, among other things, that we have the capacity to understand that recognizing the fundamental interest that human beings have in procreating implies that we need to respect that fundamental interest for those human beings who will be alive in the future and that this may put limits on our moral rights to procreate.^35^

Note that it is not claimed, and does not follow, that those who voluntarily abstain from procreating would be less than human. Rather, it is the *opportunity* to procreate that is regarded as distinctly valuable for human beings. Those who choose to abstain from procreating should (and in almost all cases will) also value the freedom to choose: it is that freedom to choose that we regard as distinctively human, that is valuable. This conceptualization of the value of procreation also allows us to understand the great sorrow experienced by those who wish to procreate but who cannot, which is not expressed in terms of not being able to access certain levels of welfare or consumer goods, but rather in terms of having to grieve over a relationship one hoped to have but does not have access to. Based on the arguments put forward by Ferracioli, one can see that this group of people is missing out on a distinct and uniquely deep and robust relationship of love.^36^

What follows? Recall that the Moral Equivalence Thesis entails that we must see procreation and overconsumption as morally equivalent in the area of distributive justice. If procreation is part of how we understand ourselves as human beings, whereas overconsumption is not, then the Moral Equivalence Thesis breaks down. The real freedom to procreate contributes to human beings seeing themselves as human beings, and hence the denial of this freedom is dehumanizing. In contrast, there is nothing dehumanizing about being denied access to overconsumption. We may argue that human beings who are living in devastating poverty are dehumanized, but this doesn’t undermine the argument, since the Moral Equivalence Thesis focuses on the alleged equivalence between procreation and overconsumption, not on the consumption needed to meet our basic needs.

One strength of the argument from human self-understanding is that it is not necessarily the case that procreative parenting is the only activity that requires scarce ecological resources. We could, for example, also make the argument that having the capability of engaging in some aesthetic activities, or enjoying beauty, is required in order to avoid dehumanization. But in contrast to the capability of procreating, there are many ways to give shape to the capability of expressing something aesthetically. Given the current ecological circumstances, this could then give us an argument for a set of ecologically-nonintrusive capabilities for aesthetic expression as entitlements of social justice. Matters are different in the case of procreative parenting, since that is a specific capability that is incommensurable with other capabilities that have a much smaller eco-footprint. In the case of procreation, there is no alternative way to realize this capability—the only way is to have a child, and not, for example, to have a plant that one can water and talk to. But it could be said that the capability is realized by having a single child, or possibly two children, depending on how much value we believe there is in the experience of having siblings. Finally, there are plenty of activities that are not required for us to understand ourselves as human beings, that is, capabilities that would lead to dehumanizing if they were not guaranteed for all. Again, which activities would fall into that category is a question that needs further argumentation, but arguably most, or perhaps even all, forms of overconsumption would be in this category.

One might argue that there are many other capabilities that have a great environmental cost that are required in order to see ourselves as human. Perhaps exploring the world and the social practices related to animal farming are examples.^37^ As mentioned in the previous paragraph, I accept that other capabilities could also be given this special status in matters of distributive justice. In fact, one might argue that we already have such a list—the list of basic human rights. And while this list includes some limited levels of mobility and certainly includes being well nourished (capabilities that can be realized in a manner that causes only limited environmental damage if one eats primarily plant-based foods), it does not include world travel, nor animal farming if there are other means of income generation and food production available. A critic who wanted to push this objection would have to give more elaborate arguments about why world travel (rather than geographically more restricted travel) and animal farming (beyond subsistence or small scale, but rather the large-scale farming that is causing the vast emissions) are needed to see oneself as human. I am very skeptical that such accounts can be given. It is correct that the argument from human self-understanding potentially opens a can of worms in that there are potentially many more activities or capabilities than procreative parenting of which one might say that denying their special nature for purposes of distributive justice could reasonably be seen as an act of dehumanization. The conclusion is that this is the debate we would need to have if we were to accept that the argument from human self-understanding should be given moral weight in judgments about distributive justice.

## Is Procreation Special?

It is now time to revisit the different parts of the argument and draw a conclusion. We started out from the Moral Equivalence Thesis, which denies that procreation has a special status in matters of distributive justice and entails that there is no morally significant difference between individuals laying claim to natural goods via overconsumption and individuals laying claim to them via procreation. Two arguments have been advanced against the Moral Equivalence Thesis. The first, the capabilitarian argument, shows that the Moral Equivalence Thesis relies on a specific underlying theory of distributive justice, namely equality of resources. The capabilitarian argument is not sufficient to reject the Moral Equivalence Thesis but does show that the acceptance of the thesis rests on an endorsement of resource egalitarianism, which is controversial. The second argument, the argument from human self-understanding, has more far-reaching consequences, since it shows why procreation and overconsumption are not morally equivalent. Procreation, and the closely related practice of parenting, are part of how we understand the human species; being given the opportunity to engage in that practice is needed as a precondition for understanding ourselves as humans. The capability of procreation is thus needed to avoid dehumanization, whereas this is not the case for overconsumption. Thus, the Moral Equivalence Thesis breaks down.

One objection to this conclusion is that it has only established that parenting is special, but not that procreation is. The objection could state that one can also meet the fundamental interest in parenting by adopting. Moreover, several philosophers have argued that we have a duty to adopt instead of raising biological children.^38^ However, there is no consensus in the literature on this matter, and some philosophers have pushed back against this claim. For example, Luara Ferracioli has developed an accounts of how procreative parenting is relevantly distinct from adoptive parenting.^39^ And Rivka Weinberg has argued against adoption being morally superior than procreative parenting because adoption is often a solution to a problem for which better solutions are available, such as enabling biological parents to care for their children.^40^ Independently of how one judges these arguments, it is important to highlight that children who would benefit from being adopted have also been created by procreative acts; so independently of what our obligations are regarding adoption, we need procreation if we want to secure the capability of parenting for all. Thus, even if those who want to parent were to have a duty to adopt, it would not undermine the capabilitarian argument or the argument from human self-understanding; rather, it would form a separate set of considerations that have to be taken into account when considering the ethics of procreation.

What follows from the rejection of the Moral Equivalence Thesis? It follows that we cannot, without an additional argument, request that parents take the natural resources that their offspring will need from their own budgets. Nevertheless, it does not follow that *all* procreative acts can be exempted in the determination of the fair allocation of ecological resources. After all, the fundamental interest for which procreation is needed is, ostensibly, the existential experiences and relationship delivered by parenting; it is not having as many babies as you like. And as several philosophers have argued, there are very good reasons why our moral right to have children is limited to protect the natural resources that will be available to future generations, natural resources that future generations will need to meet their fundamental interest in parenting or to meet their basic needs.^41^

If we endorse on the one hand the view that procreation has a special status in matters of distributive justice because it is necessary for our fundamental interest in parenting and in order to be able to see ourselves as truly human, and on the other hand the view that there are moral limits to procreation, this still leaves the unanswered question of what follows for the design of policies and institutions. This is a very difficult question to answer, and normative philosophers should collaborate with demographers, ecologists, other social scientists, and policy makers to provide answers that are relevant for the messy and unjust world in which we live.

## Footnotes

1 I will bracket the interests of nonhuman animals. A comprehensive analysis of ecological and environmental distributive ethics should include the interests of nonhuman animals and perhaps also plants. However, excluding them here does not affect my arguments.

2 On fair emission quota, see e.g. Simon Caney, “Just Emissions,” *Philosophy and Public Affairs*, Vol. 40, No. 4 (2012), pp. 255-300; Christian Baatz and Karl Ott, “In Defense of Emissions Egalitarianism?,” in Lukas Meyer and Pranay Sanklecha, eds., *Climate Justice and Historical Emissions* (Cambridge: Cambridge University Press, 2017), pp. 165-197; Olle Torpman, “The Case for Emissions Egalitarianism,” *Ethical Theory and Moral Practice*, Vol. 22 (2019), pp. 749-762; and Colin Hickey, “Climate Change, Distributive Justice, and “Pre-Institutional” Limits on Resource Appropriation,” *European Journal of Philosophy*, Early View.

3 Thomas Young “Overconsumption and Procreation: Are They Morally Equivalent?” *Journal of Applied Philosophy*, Vol. 18, No. 2, (2001), pp. 183-192; Corey MacIver, “Procreation or Appropriation?” in Sarah Hannan, Samantha Brennan and Richard Vernon, eds., *Permissible Progeny? The Morality of Procreation and Parenting* (Oxford: Oxford University Press, 2015), pp. 107-128; Paula Casal and Andrew Williams, “Rights, equality and procreation,” *Analyse und Kritik*, Vol. 17 (1995), pp. 93-108; Peter Vallentyne, “Equality and the Duties of Procreators,” in David Archard and Colin Macleod, eds. *The Moral and Political Status of Children*, (New York: Oxford University Press, 2002), pp. 195-211; Paul Bou-Habib “Parental Subsidies: The argument from insurance,” *Politics, Philosophy & Economics*, Vol. 12, No. 2, (2012), pp. 197-216; Paul Bou-Habib and Serena Olsaretti, “Equality and the Demands of Authenticity,” *Critical Review of International Social and Political Philosophy*,” Vol. 19, No. 4 (2016), pp. 434-455; Simon Caney, “Human Rights, Population, and Climate Change,” in Dapo Akande, Jaakko Kuosmanen, Helen McDermott, and Dominic Roser, eds., *Human Rights and 21*^*st*^ *Century Challenges: Poverty, Conflict and the Environment*. (Oxford: Oxford University Press, 2020), pp. 348-369.

4 Tim Meijers offers a different set of considerations in answering the question what the value of procreation is, whereby he is particularly concerned about its consistency with liberal justification. See Meijers, “The Value in Procreation: A Pro-tanto Case for a Limited and Conditional Right to Procreate,” *The Journal of Value Inquiry*, Vol. 54 (2020), pp. 627-647.

5 For this literature, see, for example, Bou-Habib, op. cit.; Bou-Habib and Olsaretti, op. cit.; Caney “Human Rights, Population, and Climate Change,” op. cit.; Elizabeth Cripps, “Climate Change, Population, and Justice: Hard Choices to Avoid Tragic Choices,” *Global Justice: Theory Practice Rethoric*, Vol. 8, No. 2 (2015), pp. 1-22; Elizabeth Cripps, “Population and Environment: The Impossible, the Impermissible and the Imperative,” in Stephen Gardiner and Allen Thompson eds., *Oxford Handbook of Environmental Ethics* (Oxford: Oxford University Press, 2016), pp. 380-390; MacIver, op. cit.; Travis Rieder, *Toward a Small Family Ethic: How Overpopulation and Climate Change are Affecting the Morality of Procreation* (New York: Springer, 2016); Vallentyne op. cit.

6 Key contributions to this literature are, among others, Colin Macleod, “Liberal Equality and the Affective Family,” in David Archard and Colin Macleod, eds., *The Moral and Political Status of Children*, (Oxford: Oxford University Press, 2002), pp. 212-230; Harry Brighouse and Adam Swift, “Parents’ Rights and the Value of the Family,” *Ethics,* Vol. 117, No. 1, (2006), pp. 80-108; Christine Overall, *Why Have Children? The Ethical Debate,* (Cambridge: MIT Press, 2012); Anca Gheaus, ‘The Right to Parent One’s Biological Baby,” *Journal of Political Philosophy,* Vol. 20, No. 4, (2012), pp. 432-455; Harry Brighouse and Adam Swift, *Family Values* (Princeton: Princeton University Press, 2014); Rivka Weinberg, *The Risk of a Lifetime: How, When, and Why Procreaton May be Permissible*, (Oxford: Oxford University Press, 2015); Anca Gheaus, “The right to parent and duties concerning future generations,” *Journal of Political Philosophy*, Vol. 24, No. 4, (2016), pp. 487-508; Tina Rulli, “Preferring a Genetically-Related Child,” *Journal of Moral Philosophy*, Vol. 13, No. 6, (2016), pp. 669-698; Luara Ferracioli, “Procreative-Parenting, Love’s Reasons and the Demands of Morality,” *The Philosophical Quarterly*, Vol. 68, No. 270, (2018), pp. 77-97.

7 Clare Heyward has criticized the Moral Equivalence Thesis on the grounds that its implementation would have unacceptable moral consequences for offspring. Yet those endorsing the Moral Equivalence Thesis could respond that they are defending it as an ethical (not political) abstract philosophical principle, and that, additionally, it remains to be discussed what would follow for all-things-considered questions of implementation, especially if that implementation concerns policies and hence would be political. See Clare Heyward, “A Growing Problem? Dealing with Population Increases in Climate Justice,” *Ethical Perspectives* Vol. 19, No. 4 (2012), pp. 703-732.

8 In this paper, it is assumed that all procreation is voluntary. A more complete analysis should include the question of how to deal with the additional complication that some procreation is not voluntary and that there is also a lot of procreation that may not be *completely* voluntary.

9 Young, op. cit; MacIver, op. cit.

10 MacIver, op. cit. p. 114.

11 Vallentyne, op. cit.; Casal and Williams, op. cit.

12 Caney, “Human Rights, Population, and Climate Change”.

13 Heyward, op cit; Cripps, op cit.

14 Ronald Dworkin, “What is Equality? Part 2: Equality of Resources,” *Philosophy & Public Affairs*, Vol. 10, No. 4 (1981), pp. 283-345.

15 This is, explicitly or implicitly, the view that can be found in most articles in this literature. For references, see footnote 3.

16 For an overview and discussion, see Paul Bou-Habib, op. cit., pp. 203-204.

17 In the case of pregnancies following from an IVF procedure, it is even more clear that some deliberate acts are needed before a pregnancy can occur.

18 John O’Neill, “The Overshadowing of Needs,” in Frederik Rauschmayer, Ines Omann and Johannes Frühmann, eds. *Sustainable Development. Capabilities, Needs, and Well-Being*, (London: Routledge, 2011), pp. 25-42

19 Elizabeth Anderson, “What’s the point of equality?” *Ethics* Vol. 109 (1999), pp. 287-337; Roland Pierik and Ingrid Robeyns, “Resources versus Capabilities: Social Endowments in Egalitarian Theory,” *Political Studies*, Vol. 55, No. 1 (2007), pp. 133-152.

20 Bou-Habib, op. cit.; Bou-Habib and Olsaretti, op. cit.

21 For a discussion of the methodological difference between moral analysis based on theories versus based on principles and values, see Jonathan Wolff, “Method in Philosophy and Public Policy: Applied Philosophy versus Engaged Philosophy,” in Annabel Lever and Andrei Poama, eds., *The Routledge Handbook of Ethics and Public Policy* (London: Routledge, 2018), pp. 13-24.

22 As is done in an unpublished companion paper, entitled “Sustainable procreative freedom”, which investigates morally permissible population policies in an ecologically unsustainable world.

23 Serena Olsaretti, “Children as negative externalities?” *Politics, Philosophy & Economics*, Vol. 16, No. 2, (2017), pp. 152-173.

24 Amartya Sen, “Well-being, Agency and Freedom: the Dewey Lectures 1984”, *Journal of Philosophy* (1985), pp. 169-221; Martha Nussbaum, *Frontiers of Justice,* (Cambridge, Mass.: The Belknap Press of Harvard University Press, 2006); Ingrid Robeyns, *Wellbeing, Freedom and Social Justice. The Capability Approach Re-examined,* (Cambridge: Open Book Publishers, 2017).

25 See, for example, Amartya Sen, *The Idea of Justice,* (London: Allen Lane, 2009); Nussbaum, op. cit.; Harry Brighouse and Ingrid Robeyns, eds., *Measuring Justice: Primary Goods and Capabilities*, (Cambridge: Cambridge University Press, 2010).

26 For a discussion on why parenting should not simply be equated with unconditional love, see Christine Overall, op. cit., pp. 212-214.

27 Macleod, op. cit.

28 Brighouse and Swift, op. cit.

29 Brighouse and Swift, “Parents’ Rights and the Value of the Family,” p. 96.

30 Overall, op. cit., pp. 209-220.

31 Ferracioli, op. cit., pp. 83-84.

32 Weinberg, op. cit., pp. 36-42.

33 However, note that from the fact that there are moral limits it does not follow, without further arguments, that there should be political or legal limits; that would be a further claim requiring additional arguments.

34 Conly argues we are morally permitted to one child per couple; Overall argues for a limit of one child per person. See Conly, op. cit., p. 49-57; Overall op. cit., 173-184.

35 See, e.g., Overall, op. cit.; Conly, op. cit.; Gheaus, “The right to parent and duties concerning future generations”; Tim Meijers, “Climate change and the right to one child” in Gerard Bos and Marcus Düwell, eds., *Human Rights and Sustainability*, (London: Routledge, 2016), pp. 181-194.

36 Ferracioli, op. cit.

37 I thank an anonymous referee for raising this objection.

38 For example, Daniel Friedrich, “A Duty to Adopt?,” *Journal of Applied Philosophy* Vol. 30, No. 1, (2013), pp. 25-39; Rulli op. cit..

39 Ferracioli, op. cit.

40 Weinberg, op. cit., pp. 43-33.

41 See, among others, Overall, op. cit.; Conly, op. cit.; Gheaus, “The right to parent and duties concerning future generations”; Meijers, “Climate change and the right to one child,”; Cripps, “Climate Change, Population, and Justice”; and Rieder, op. cit.

